# Chemosymbiotic bivalves from the mud volcanoes of the Gulf of Cadiz, NE Atlantic, with descriptions of new species of Solemyidae, Lucinidae and Vesicomyidae

**DOI:** 10.3897/zookeys.113.1402

**Published:** 2011-06-28

**Authors:** Graham Olive, Clara F. Rodrigues, Marina R. Cunha

**Affiliations:** 1 BioSyB, National Museum of Wales, Cathays Park, Cardiff, CF10 3NP, Wales, UK; 2 CESAM, Departamento de Biologia, Universidade de Aveiro, Campus Universitário de Santiago, 3810–193 Aveiro, Portugal

**Keywords:** Bivalvia, chemosymbiotic, taxonomy, Gulf of Cadiz

## Abstract

The chemosymbiotic bivalves collected from the mud volcanoes of the Gulf of Cadiz are reviewed. Of the thirteen species closely associated with chemosynthetic settings two Solemyidae, *Solemya (Petrasma) elarraichensis* **sp. n.** and *Acharax gadirae* **sp. n.**, one Lucinidae, *Lucinoma asapheus* **sp. n.**, and one Vesicomyidae, *Isorropodon megadesmus* **sp. n.** are described and compared to close relatives of their respective families. The biodiversity and distribution of the chemosymbiotic bivalves in the Gulf of Cadiz are discussed and compared to the available information from other cold seeps in the Eastern Atlantic and Mediterranean. Although there is considerable similarity at the genus level between seep/mud volcano fields in the Eastern Atlantic and Mediterranean, there is little overlap at the species level. This indicates a high degree of endemism within chemosymbiotic bivalve assemblages.

## Introduction

Chemosynthetic bivalves are prominent constituents of the fauna of cold seeps and are represented in that setting by five families: Solemyidae, Lucinidae, Vesicomyidae, Thyasiridae and Mytilidae (Sibuet and Olu 1998, Sibuet and Olu-Le Roy 2002, Sahling et al. 2003, Levin 2005). Recently the presence of bacteria in the gills in species of *Nucinella* and *Huxleyia* has been demonstrated (Oliver and Taylor in preparation), confirming the previous inclusion of the Manzanellidae in this group (Cosel and Bouchet 2008).

The occurrence of chemosymbiotic bivalves in the extensive mud volcano fields of the Gulf of Cadiz was first reported by Pinheiro et al. (2003). Then followed a series of more in-depth studies: on polychaete commensals of solemyid hosts (Ravara et al. 2007); on the distribution and taxonomy of Thyasiridae (Rodrigues et al. 2008); on the phylogenetic relationships of *Bathymodiolus mauritanicus* Cosel (Génio et al. 2008) and on the molecular characterization of chemosymbiotic endosymbionts of solemyids, lucinids, thyasirids and mytilids (Rodrigues et al. 2010, Rodrigues and Duperron 2011).

Chemosynthetic bivalve faunas have been discovered elsewhere in the Eastern Atlantic, notably off tropical West Africa (Cosel and Salas 2001, Olu-Le Roy et al. 2007, Cosel and Olu 2009) and in the eastern Mediterranean (Salas and Woodside 2002, Olu-Le Roy et al. 2004, Carlier et al. 2010). For the Vesicomyidae, at the species level, these faunas are considered disparate (Cosel and Salas 2001, Krylova and Sahling 2010). Conversely studies on *Bathymodiolus* suggest that the species occurring in the Gulf of Cadiz is not only found along the West African margin but is amphi-Atlantic (Génio et al. 2008). The biogeographic patterns within these faunas are currently unresolved and require further taxonomic characterization, which this paper begins to address.

This paper intends to provide the taxonomic basis for the chemosynthetic bivalves in the Gulf of Cadiz and includes the description of two new species of Solemyidae, one new species of Lucinidae and one new species of Vesicomyidae. Notes on the biogeography of these taxa in the Atlantic are given with special emphasis on the relationships between the Eastern Mediterranean, Gulf of Cadiz and West Africa.

## Materials and methods

### Study area.

**Figure 1.**
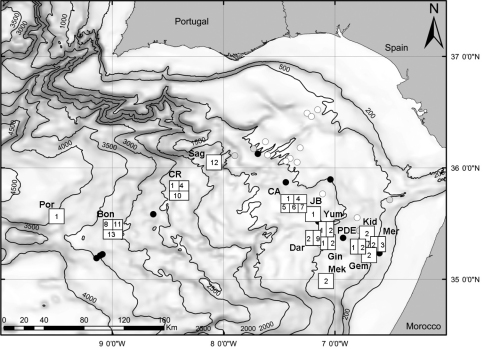
Map of the study area (Gulf of Cadiz) and location of sampling sites. **squares with numbers**, mud volcanoes with chemosymbiotic bivalves: **full black circles**, mud volcanoes visited during the study but bivalves not found: **grey circles**: mud volcanoes and other structures not visited during the study. **Bon**, Bonjardim MV; **CA**, Captain Arutyunov MV; **CR**, Carlos Ribeiro MV; **Dar**, Darwin MV; **Gem**, Gemini MV; **Gin**, Ginsburg; **JB**, Jesus Baraza MV; **Kid**, Kidd MV; **Mek**, Mèknes MV; **Mer**, Mercator MV; **PDE**, Pen Duick Escarpment; **Por**, Porto MV; **Sag**, Sagres MV; **Yum**, Yuma MV. The numbers inside the squares indicate the presence of the following species. **1** *Acharax gadirae* **2** *Petrasma elarraichensis* **3** *Lucinoma asapheus* **4** *Thyasira vulcolutre* **5** *Spinaxinus sentosus* **6** *Isorropodon megadesmus* **7** *Isorropodon* sp. indet. **8** *Christineconcha* cf. *regab* **9** *Bathymodiolus mauritanicus* **10** *Idas* sp. **11** *Laubiericoncha chuni* (empty shells only) **12** *Callogonia cyrili* (empty shells only) **13** *Pliocardia* sp. (empty shells only).

The Gulf of Cadiz is located in the NE Atlantic Ocean between 34°N and 37°15'N and 6°W to 9°45'W. It is enclosed by the southern Iberian and northern Moroccan margins, west of Gibraltar Strait. The geological history of the Gulf of Cadiz is intimately related to plate tectonic interaction between Southern Eurasia and North Africa and is driven by two major mechanisms: a) subduction associated with the westward emplacement of the Gibraltar Arc and formation of the Gulf of Cadiz accretionary wedge, probably not active at present and b) oblique lithosphere collision between Iberia and Nubia, active at present and causing active thrusting (Zitellini et al. 2009). It is now well established that the whole area is under compressive deformation and that mud volcanism and processes associated with the escape of hydrocarbon-rich fluids sustain a broad diversity of chemosynthetic assemblages. This extensive area encompasses over forty mud volcanoes (here after as MV), at depths ranging from 200 to 4000m (confirmed by coring) (Mazurenko et al. 2002, Pinheiro et al. 2003, Magalhães 2007), and active methane seepage has been documented on several locations (Kenyon et al. 2000, Gardner 2001, Niemann et al. 2006, Stadnitskaia et al. 2006, Hensen et al. 2007). Biological samples were available from 30 sites but for this study only thirteen MVs and one structure (PDE) yielded chemosymbiotic bivalves (Fig. 1).

In the shallow Moroccan margin the El Arraiche field encompasses Renard Ridge (including Pen Duick Escarpment), Vernadsky Ridge and several mud volcanoes (e.g. Al Idrisi, Mercator, Fíuza, Gemini, Kidd MVs) located at depths from 200 to approximately 600m depth: The proximity to the euphotic zone and to the African coast adds to the great productivity observed in the area. Dead cold-water scleractinean coral reefs, carbonate crusts and exposed carbonate chimneys characterize the Renard and Vernadsky Ridges. Carbonate crusts, rock blocks and clasts are often found in the craters of the shallow mud volcanoes where mild seepage activity has been recorded (Van Rensbergen et al. 2005). Mercator MV, one of the shallowest mud volcanoes differs significantly from the other mud volcanoes by the high chloride enrichment of its pore water (Van Rensbergen et al. 2005). The top of Mercator MV shows patches of disturbed sediments from which gas venting is occasionally observed. Solitary corals (*Caryophyllia* sp.), accompanied by Cidaridae echinoids and Onuphidae polychaetes (*Hyalinoecia*) are the most conspicuous organisms seen during video surveys of the Mercator MV crater.

The western Moroccan field comprises several mud volcanoes (e.g. Meknès, Student, Yuma, Ginsburg, Jesus Baraza, Darwin MVs) at intermediate depths (700–1200m) located along an extensive province of carbonate and mostly dead cold-water coral mounds. The widespread presence of authigenic carbonates and also extensive *Neptunea* and *Bathymodiolus* graveyards (usually within the crater of the mud volcanoes) suggest that this was a very active seepage area in the past. Darwin MV differs from the others in this area because its crater is completely covered by large carbonate slabs and crusts. The fissures among slabs and depressions with scattered crust are filled with abundant shell ash and occasionally small clumps of living *Bathymodiolus mauritanicus* Cosel, 2002. Meknès MV is the southernmost Moroccan mud volcano rising isolated among an extensive field of small coral mounds. The crater is formed by stiff, sometimes heavily disturbed, green mud breccia with scattered clasts and a striking large number of empty shells of the gastropod *Neptunea*. Except for a few *Paromola* individuals, living megafauna is rarely sighted in the crater

The deep-water field (1300–4000m), mostly within the Portuguese margin includes several mud volcanoes (e.g. Captain Arutyunov, Carlos Ribeiro, Bonjardim and Porto MVs) that are aligned along major crustal strike–slip faults associated with the African-Eurasian plate boundary (Duarte et al. 2005). Gas hydrates were recovered from these mud volcanoes and the methane concentrations yield the highest records from the Gulf of Cadiz (Kenyonet al. 2000, 2001, 2002, 2003, 2006; Akhmetzhanov et al. 2007, 2008). Video surveys of these deeper mud volcanoes often show conspicuous siboglinid fields (e.g. Porto MV) in the active craters and exuberant sponge and gorgonian patches at the crater rim and upper flank (e.g. Carlos Ribeiro MV).

### Sampling.

Samples were collected between 2002 and 2006 during TTR (Training Through Research) 12, TTR 14, TTR15 and TTR16 cruises onboard RV Prof. Logachev and MSM.01-03 cruise onboard RV Maria S. Merian (IFM–GEOMAR). The material was collected using TV-assisted grabs or USNEL box-corers. Occasionally faunal specimens were also recovered from Reineck box-corer, multiple corer or lander samples that were carried out for different purposes. Whenever possible the specimens were sorted onboard and preserved in 70 or 96% ethanol (the latter preserved for molecular analysis).

### Deposition of samples.

The majority of specimens are deposited in the Biological Research Collection of the Department of Biology, University of Aveiro but the holotype; some paratypes and selected specimens are deposited in the National Museum of Wales.

### Institutional abbreviations.

DBUADepartment of Biology, University of Aveiro (Biological Research Collection); IFM–GEOMARInstitut für Meereskunde - Forschungszentrum für marine Geowissenschaften; IOC–UNESCOIntergovernmental Oceanographic Commission – United Nations Educational, Scientific and Cultural Organization; NMW.ZNational Museum of Wales, Cardiff, Great Britain.

### Measurements.

All measurements were made using Sylvac™ vernier calipers accurate to 0.01mm but are given to the nearest tenth.

## Systematics

### Order Solemyoida Dall, 1889 Superfamily Solemyoidea Gray, 1840 Family Solemyidae Gray, 1840

Solemyids are among the most ancient bivalves dating from the Paleozoic (Métivier and Cosel 1993). They are protobranch in organization and characterized by an elongate shell (up to 220mm) with a posteriorly situated toothless hinge. The thick brown periostracum is much larger than the calcified part of the valve and is folded inwards by the muscular mantle edge upon closing of the valves (Métivier and Cosel 1993).

Solemyidae taxonomy is complex. Taylor et al. (2008) and Kamenev (2009) recognized two extant genera, *Solemya* and *Acharax*, with a further four subgenera within *Solemya*, namely *Petrasma*, *Austrosolemya*, *Solemyarina* and *Zesolemya*. Solemyids, other than *Acharax* are generally found at continental shelf and upper-slope depths (0 to 600m), although Solemya (*Petrasma*) *pervernicosa* has been recorded at 1500m (Kamenev 2009). *Acharax* is generally restricted to deep-sea settings from ~400m on the continental slope to the deepest sites of the Japan Trench (Neulinger et al. 2006). Shallow dwelling solemyids live in sediments with high organic matter content, often at reduced oxygen concentrations. Hydrogen sulphide is frequently present due to sulphate reduction coupled with organic matter degradation (Conway et al. 1992). In contrast, the genus *Acharax* has been recovered from cold seep locations (see review by Sibuet and Olu 1998) and in sediments influenced by hydrothermal venting (Juniper et al. 1992, Métivier and Cosel 1993). *Acharax* species are morphologically similar but molecular data suggests a degree of cryptic speciation (Neulinger et al. 2006).

Superficially, all solemyids appear so similar that specimens discovered at various deep-sea sites might have been misclassified as *Solemya* (see review by Sibuet and Olu 1998).

### Genus Solemya Lamarck, 1818

#### Subgenus Petrasma

Dall, 1908

##### Type species.

*Solemya borealis* Totten, 1834

##### Definition.

As given by Taylor et al. 2008. Ligament wholly internal, supported by a buttress and lacking posterior or lateral extensions.

#### Solemya (Petrasma) elarraichensis sp. n.

urn:lsid:zoobank.org:act:32A6013D-4378-462A-BABA-4AFC4D26FB1E

http://species-id.net/wiki/Solemya_(Petrasma)_elarraichensis

Figs 2 3A–D 4

##### Material examined.

*Holotype*: one specimen, TTR14, stn AT528GR, El Arraiche field, Kidd MV, 35°25.304'N, 06°43.972'W, 489m, 03 August 2004, NMWZ.2010.4.1

*Paratypes*: ten specimens, same data as holotype, NMWZ.2010.4.2; seven specimens, TTR15, stn AT569GR, El Arraiche field, Mercator MV, 35°17.917'N, 06°38.717'W, 358m, 25 July 2007, DBUA.

*Other material examined*: eight juveniles specimens, same data as holotype; two specimens, TTR12, stn AT407GR, El Arraiche field, Pen Duick Escarpment, 35°17.695'N, 06°47.082'W, 560m, 15 July 2002; three specimens, TTR14, stn AT560B, El Arraiche field, Kidd MV, 35°25.306'N, 06°43.976'W, 498m, 8 August 2004; one specimen, TTR15, stn AT586GR, Western Moroccan field, Meknès MV, 34°59.146'N, 07°04.380'W, 701m, 28 July 2005; four specimens, TTR16, stn AT604GR, Western Moroccan field, Yuma MV, 35°25.820'N, 07°06.330'W, 1030m, 29 May 2006; two specimens, TTR16, stn AT607GR, Western Moroccan field, Ginsburg MV, 35°22.677'N, 07°04.979'W, 983m, 29 May 2006.

##### Measurements (in mm)

**Table:** 

	Station	Length	Height	Posterior Length
Holotype	AT528GR	33.8	14.1	9.8
Paratype	AT528GR	29.2	10.5	8.0
Paratype	AT528GR	25.6	10.0	6.2
Paratype	AT528GR	23.1	8.4	6.0
Paratype	AT528GR	14.7	5.3	3.6
Paratype	AT528GR	22.0	7.9	6.0
Paratype	AT528GR	11.6	4.7	2.5
Paratype	AT528GR	17.6	6.8	5.2

##### Description.

**Figure 2.**
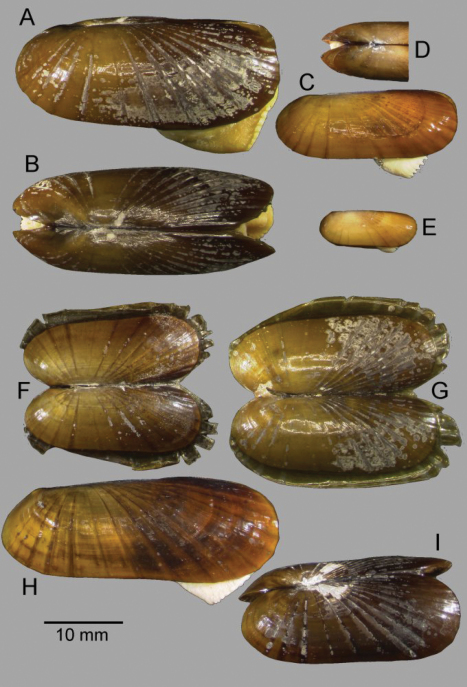
*Solemya (Petrasma) elarraichensis* sp. n. **A–E** from Kidd MV; **A–B** lateral and dorsal views of holotype **C–D** lateral and dorsal views of medium sized paratype **E** lateral view of small paratype. **F** paired valves from Pen Duick Escarpment **G** paired valves from Mercator MV **H** lateral view of specimen from Meknès MV **I** lateral view of shell from Yuma MV.

**Figure 3. A–F:**
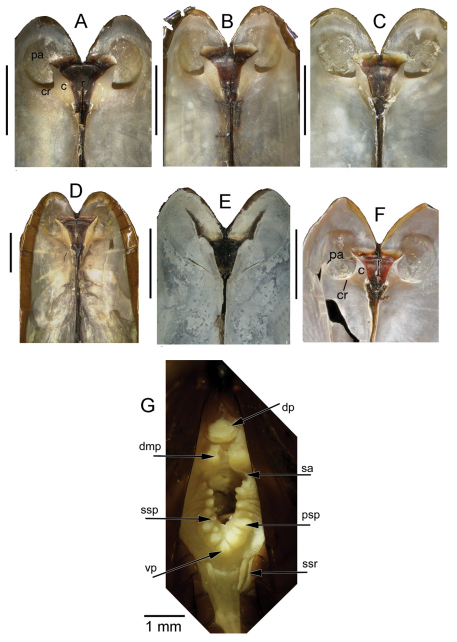
Internal views of ligament, scale bars = 5mm. **A–D** *Solemya (Petrasma) elarraichensis* sp. n. from **A** Kidd MV **B** Pen Duick Escarpment **C** Mercator MV **D** Yuma MV. **E** *Solemya togata*, Mediterranean **F** *Solemya (Petrasma) velum*, Rhode Island (from Taylor et al. 2009). c, chondrophore; cr chondrophore ridge; pa, posterior adductor scar; r, resilium. **G** posterior siphon of *Solemya (Petrasma) elarraichensis*. dp, dorsal papilla; dmp, dorsal marginal papillae; psp, primary siphonal papillae; sa, smooth area; ssp, secondary siphonal papillae; ssr, subsiphonal ridge; vp, ventral papilla.

*Shell* (Figs 2, 3): to 35mm in length. Fragile. Equivalve. Inequilateral, beaks situated at 1/4 length of shell from posterior margin. Outline subcylindrical, compressed, length about 2.6 times height, slightly deeper towards the anterior, dorsal and ventral margins subparallel, anterior margin more broadly rounded than anterior, posterior dorsal margin projecting a little. Beaks indistinct, umbos sunken. Hinge teeth absent. Ligament primarily internal, supported by a prominent chondrophore that extends only slightly as a chondrophore ridge around the posterior adductor, lacking posterior and anterior extensions but a small roughly heart shaped area is present in front of the chondrophore and this is also visible externally just behind the beaks. Periostracum persistent and extending well beyond the shell margin, initially yellowish brown in colour but darkening with growth to a dark chestnut brown. Sculpture of weak radial ridges, 5–6 over the posterior and 10–12 over median and anterior. Adductor scars impressed, dorsal part of posterior scar angulate where bounded by chondrophore ridge, anterior adductor scar larger, spatulate in outline.

**Figure 4.**
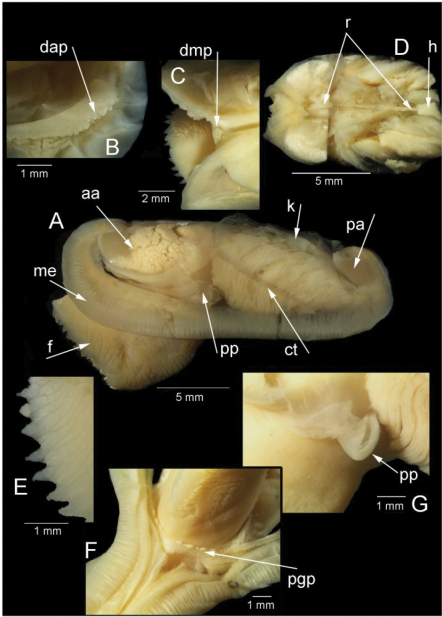
*Solemya (Petrasma) elarraichensis* sp. n., Pen Duick, stn. AT407GR, 560m. Anatomy. **A** whole animal viewed from left side **B** papillae on dorsal anterior mantle edge **C** single, large papilla in dorsal median position **D** posterior dorsal dissection showing rectum passing through heart **E** marginal papillae on foot **F** papillae on mantle edge surrounding pedal gape **G** palp. aa, anterior adductor muscle. ct, ctenidium. dap, dorsal anterior papillae. dmp, dorsal median papilla. f, foot. h, heart. k, kidney. me, mantle edge. pa, posterior adductor muscle. pgp, papillae surrounding pedal gape. pp, palp. r, rectum.

*Anatomy* (Figs 3G, 4): The posterior siphonal opening is surrounded by a series of papillae: A single large dorsal papilla (dp) lies above two smaller but still large papillae (dmp) on the dorsal margin of the opening, below these is a short smooth section (sa) followed by a series of papillae increasing in size towards the ventral margin, there are 6 primary papillae (psp) on either side and a single ventral median papillae, between these on the inner side are smaller papillae (ssp); a pair of subsiphonal ridges (ssr) are present below the siphonal crown.

The mantle edge is fused from the posterior siphon for half the length of the ventral margin where there is a large anterior pedal gape. The mantle edge surrounding the rear of the foot bears a few tabulate papillae (pgp). The anterior dorsal mantle edge is prominently papillate (dap) and there is a single papilla on the junction of the mantle edge anterior of the anterior adductor muscle (admp). The foot is very large with a broad oval sole, this fringed by large papillae, all equal in size. The ctenidium is large with numerous laminar filaments attached to a prominent gill axis. The palps are short, twisted and flattened with cup shaped terminations. The gut is present but difficult to examine due to its small dimensions but the hind gut and rectum were easily visible.

##### Distribution.

*Solemya (Petrasma) elarraichensis* is presently only known from the mud volcano fields in the Gulf of Cadiz, Eastern Atlantic. The majority of specimens have been taken from the El Arraiche field off the coast of Morocco in Kidd, Fíuza and Mercator MVs and the Pen Duick Escarpment at depths between 358–560m. A few specimens have been taken from the Western Moroccan field at the Meknès, Yuma, Ginsburg and Darwin MVs at the slightly deeper range of 700–1115m.

##### Etymology.

*elarraichensis*, denoting the geographic origin of the type locality; the El Arraiche field.

##### Remarks.

The form of the ligament, which is primarily internal, supported by a chondrophore and lacks any lateral or anterior extensions, confirms the placement of *Solemya (Petrasma) elarraichensis* in the subgenus *Petrasma* Dall, 1908 (Taylor et al. 2008). Some of the specimens carry an initial identification of “cf. *Solemya togata*” as might be expected from the proximity to the Mediterranean but the ligament of *Solemya s.s.* has prominent anterior extensions (Fig. 3E).

The subgenus *Petrasma* is not known from the North-East Atlantic but is represented in the Western Atlantic by three species. Two species are known from near shore waters off the northeast coast of the USA: *Solemya (Petrasma) velum* (Say, 1822) and *Solemya (Petrasma) borealis* (Totten, 1834). The third, *Solemya (Petrasma) occidentalis* (Deshayes 1857) is known from the warm waters of Florida, Caribbean and S. America (Mikkelsen and Bieler 2008).

We note that the curvature of the chondrophore and chondrophore ridge is circular in *Solemya (Petrasma) velum* (Fig. 3F) but angular in *Solemya (Petrasma) elarraichensis* (Figs 3A–D). Furthermore, the siphonal papillae of *Solemya (Petrasma) velum* are by comparison less in number and reduced in development (Morse 1913, Taylor et al. 2008).

Abbott (1974) following Morse (1913) noted that the siphon of *Solemya (Petrasma) borealis* differed markedly from that of *Solemya (Petrasma) velum*, confirming that siphonal characters were important for distinguishing species. In *Solemya (Petrasma) borealis*, the ventral-most dorsal marginal papillae are very large, and as big as the dorsal papilla, and much larger than any of the ventral papillae. This contrasts with the condition in *Solemya (Petrasma) elarraichensis* where the ventral-most dorsal marginal papillae are smaller than the dorsal papilla and where the ventral papillae are fewer in number and distinctly increasing in size ventrally, with the ventral-most papillae equal in size to the dorsal marginal papillae. Conway et al. (1992), following Barnard (in Reid 1980) suggested that *Solemya (Petrasma) borealis* lacked a gut and this would be in contrast with *Solemya (Petrasma) velum* and *Solemya (Petrasma) elarraichensis*.

The character of the ligament and chondrophore are rather similar in *Solemya (Petrasma) elarraichensis* and *Solemya (Petrasma) borealis*.

Abbott (1974) and Mikkelsen and Bieler (2008) note that *Solemya (Petrasma) occidentalis* lacks any perceptible chondrophore ridge and give this as the main characteristic separating *Solemya (Petrasma) occidentalis* from *Solemya (Petrasma) velum* and, therefore, also from *Solemya (Petrasma) elarraichensis*.

Ecologically *Solemya (Petrasma) velum* and *Solemya (Petrasma) borealis* are very different from *Solemya (Petrasma) elarraichensis* in that they are not associated with deep-water methane seeps. In contrast they are found in sublittoral or shallow shelf settings with high organic enrichment (Morse 1913 in Conway et al. 1992). Mikkelsen and Bieler (2008) give a similar habitat for *Solemya (Petrasma) occidentalis* (Deshayes, 1857) noting its occurrence in mangrove channels and around sewage outfalls.

Given the above differences in habitat and form we conclude that none of the Atlantic species is amphi-Atlantic, unlike *Solemya (Petrasma) pervernicosa* Kuroda, 1948, which is considered to be amphi-Pacific by Kamenev (2009). If any of the Western Atlantic species were amphi-Atlantic it is unclear why, in the Eastern Atlantic, they should be absent from their typical settings (which are plentiful) and found only in deep water methane seeps.

Other North Atlantic species referred to as *Solemya*, *Solemya grandis* Verrill and Bush, 1898 and *Solemya caribbaea* Vokes, 1970 are excluded here because both belong to the genus *Acharax* (Abbott 1974).

A solemyid living at a pockmark, at a depth of 1607m, has been reported from the Eastern Mediterranean (Rodrigues et al. 2011). Unfortunately the small, single specimen was damaged and its taxonomic affinities remain unclear.

In conclusion, there are sufficient morphological and ecological grounds for considering the Gulf of Cadiz species of *Petrasma* to be new to science.

#### Genus Acharax

Dall, 1908

##### Type species:

*Solemya johnsoni* Dall, 1891

##### Definition.

As given by Taylor et al. 2008. Ligament external, as a high arched band.

#### Acharax gadirae sp. n.

urn:lsid:zoobank.org:act:A2467D10-D03D-4271-9993-0C2A6CD22944

http://species-id.net/wiki/Acharax_gadirae

Figs 5– 6

##### Type material.

*Holotype*: one specimen, TTR12, stn AT391GR, Western Moroccan field, Jesus Baraza MV, 35°35.439'N, 07°12.264'W, 1105m, 09 July 2002, NMWZ.2010.4.3.

*Paratypes*: one specimen, same data as holotype, DBUA; one shell, TTR 12, stn AT392G, deep-water field, Captain Arutyunov MV, 35°39.658'N, 07°20.018'W, 1320m, 9 July 2002, DBUA; one shell, TTR 16, stn AT607GR, Western Moroccan field, Ginsburg MV, 35°22.677'N, 07°04.979'W, 983m. 29 May 2006, NMWZ.2010.4.4.

*Other material examined:* one specimen, TTR16, stn AT602GR, El Arraiche field, Pen Duick Escarpment, 35°17.693'N, 06°47.089'W, 556m, 28 May 2006; one specimen, TTR16, stn AT604GR, Western Moroccan field, Yuma MV, 35°25.820'N, 07°06.330'W, 1030m, 29 May 2006; one specimen, TTR16, stn AT605GR, same locality, 35°25.046'N, 07°05.450'W, 975m, 29 May 2006; one specimen, TTR16, stn AT615GR, deep-water field, Carlos Ribeiro MV, 35°47.238'N, 08°25.272'W, 2200m, 31 May 2006; one specimen, TTR16, stn AT617K, same locality, 35°47.246'N, 08°25.303'W, 2230m, 31 May 2006; two specimens, MSM01.03, stn 145, deep-water field, Porto MV, 35°33.773'N, 09°30.416'W, 3902m, 3 June 2006.

##### Measurements (in mm)

**Table:** 

	Station	Calcified Shell Length	Calcified Shell Height	Calcified ShellPosterior Length	Actual length	Anterior Ribs/Posterior Ribs
Holotype	AT391GR	56.3	21.1	14.5	59.5	9/4
Paratype	AT391GR	60.0	22.0	14.2	65.4	8/4
Paratype	AT392GR	67.0	25.0	19.1	85.0	8/4
Paratype	AT607 GR	42.8	15.9	10.6	48.9	9//4

##### Description.

**Figure 5. A–F:**
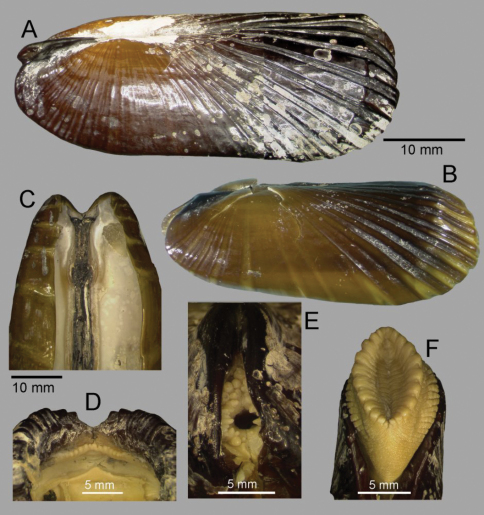
*Acharax gadirae* sp. n. **A** Holotype, stn. AT391GR, Jesus Baraza MV **B** Paratype, stn. AT607GR, Ginsburg MV **C** interior view of posterior ligament, stn. AT392GR, Jesus Baraza MV **D** anterior dorsal mantle edge, st. AT391GR **E** posterior siphon, st AT391GR **F** foot, stn. AT391GR

(Fig. 5) Calcified shell to 67mm in length, to 85mm including periostracal fringe. Robust. Equivalve. Inequilateral, beaks situated at 1/4 length of shell from posterior margin. Outline subcylindrical, compressed, calcified shell length about 3 times height, slightly deeper towards the anterior, dorsal and ventral margins subparallel, anterior margin more broadly rounded than anterior, posterior dorsal margin projecting a little. Including periostracal fringe, anterior appears greatly expanded compared with posterior. Beaks indistinct, umbos sunken. Hinge teeth absent. Ligament external, as a high arched band posterior of the beaks and supported by a thickened shell margin; an oval area of ligament is present immediately behind the beaks and visible internally, anterior of the beaks shell margins fused by periostracal material along entire dorsal margins. Periostracum persistent and extending well beyond the shell margin, initially yellowish brown in colour but darkening with growth to dark brown and black; periostracal frill thickened over ribs but entire. Sculpture of radial ridges, 4 closely spaced over the posterior; median area almost smooth with 2–3 low ribs; anterior with 8–9 deeply cut ribs. Adductor scars impressed, posterior scar subcircular, anterior adductor scar larger, spatulate in outline. Anterior inner shell margin scalloped corresponding to radial ribs.

Posterior siphonal opening surrounded by a series of papillae (Fig. 5E): A single large dorsal papilla (dp) lies above 2–3 pairs of slightly smaller papillae (dmp) on the dorsal margin of the opening, below these surrounding the opening is a series of approximately alternating large and small papillae with those most ventral the largest.

The mantle edge is fused from the posterior siphon for half the length of the ventral margin where there is a large anterior pedal gape. The mantle edge surrounding the rear of the foot is papillate. The anterior dorsal mantle edge is prominently papillate (Fig. 5D). The foot is very large with a broad oval sole, the margin interdigitates between large and small blunt papillae. The ctenidium is large with numerous laminar filaments attached to a prominent gill axis. The palps are short, twisted and flattened with cup shaped terminations. The presence or absence of a gut could not be confirmed.

##### Distribution.

*Acharax gadirae* is presently only known from the mud volcano fields in the Gulf of Cadiz, Eastern Atlantic. The specimens have been taken from the Western Moroccan field at Yuma, Ginsburg and Jesus Baraza MVs, and from the deep-water field at Captain Arutyunov, Carlos Ribeiro and Porto MVs at depths between 975 to 3902m. A single specimen was recovered from the shallower El Arraiche field in Pen Duick Escarpment at 556m.

##### Etymology.

*gadirae*, from the Phoenician “Gadir” the original name for Cadiz and meaning “walled fortification” and also the root of many Moroccan names such as Agadir. Named to indicate the widespread range across the Moroccan and Iberian margins.

##### Remarks.

The genus *Acharax* is recognizable from the large external ligament and the generic placement of *Acharax gadirae* is confirmed.

The genus is rare in the Atlantic Ocean unlike the situation in the Pacific where species of *Acharax* are frequently recorded from chemosynthetic settings (Neulinger et al. 2006). Only two species are known from the Atlantic. *Acharax grandis* (Verrill and Bush 1898) is known only from the original material collected from depths between 548 and 2926m in the region of the New York Bight. *Acharax caribbaea* (Vokes 1970) again is only recorded from the original material collected from a depth of 350m off Colombia in the Caribbean Sea. However, the genus is recorded in recent studies from both the Gulf of Mexico (Carney 1994) and the Barbados prism (Olu et al. 1997) but the species are not identified.

*Acharax grandis* differs from both *Acharax gadirae* and *Acharax caribbaea* in being less inequilateral with the beaks distinctly more towards the mid-line. *Acharax caribbaea* differs from both *Acharax gadirae* and *Acharax grandis* in having very few (4) anterior ribs compared with the 6–8 on *Acharax grandis* and 8–9 on *Acharax gadirae* of similar size. Unfortunately, there are no anatomical data for either *Acharax grandis* or *Acharax caribbaea*, making a thorough comparison impractical.

There are no given ecological data for either *Acharax grandis* or *Acharax caribbaea*. The type locality for *Acharax grandis*, which is the region around the Hudson Shelf and Canyon, has no recorded seep or vent activity. In contrast the region around the type locality of *Acharax caribbaea* is known for a variety of chemosynthetic settings (Carney 1994).

**Figure 6.**
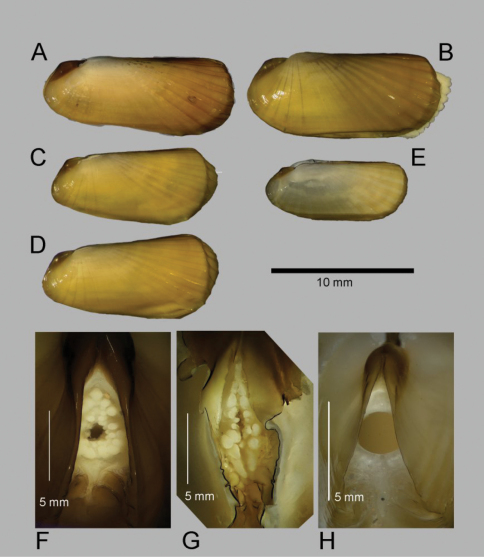
*Acharax gadirae* sp. n. **A** stn. AT602GR, Pen Duick Escarpment **B** stn. 145, Porto mud volcano **C–D** stns AT617GR & AT61GR, Carlos Ribeiro mud volcano **E** stn. 199, Capt Arutyunov mud volcano. **F–E** posterior siphon **F** specimen A **G** specimen D **H** specimen E.

The bathymetric range of *Acharax* in the Gulf of Cadiz is large, 556–3902m and specimens have been taken at many mud volcanoes raising the possibility that more than one species is involved. Unfortunately the specimens from the abyssal sites are all small about 10mm or less making comparison with the large specimens from the bathyal sites inconclusive. The specimens from Carlos Ribeiro MV (2200m) (Fig. 6C–D) are prominently wedge shaped in outline compared with the specimen from Porto MV (3902m) (Fig. 6B). The latter is not dissimilar to those from Capt. Arutyunov MV (1325m) (Fig. 6E) with the specimen from Pen Duick Escarpment (556m) (Fig. 6A) somewhat more elongate but not as wedge shaped as those from Carlos Ribeiro MV.

The specimens from the Capt Arutyunov MV are most problematic in that the siphonal opening appears to be devoid of any surrounding tentacles or papillae (Fig. 6H). This does not appear to be a function of size as similar specimens from other sites have siphonal papillae. Should this observation be confirmed in further material it would be appropriate to describe this as a separate species. Comparing the siphonal papillae of specimens from Pen Duick Escarpment (Fig. 6F) and Carlos Ribeiro MV (Fig. 6G) indicates a more complex arrangement in the latter but, with so few specimens, this is inconclusive.

### Superfamily Lucinoidea Fleming, 1828 Family Lucinidae Fleming, 1828

The Lucinidae is, by far, the most disparate and species-rich family of chemosymbiotic bivalves and are thoroughly reviewed by Taylor and Glover (2006). Although they occupy a wide range of habitats, they are relatively infrequent in deep-sea settings (Taylor and Glover 2009). Some species are associated with cold seeps and mud volcanoes, oxygen minimum zones and a single species is known from a hydrothermal vent (Taylor and Glover 2006). Of most frequent occurrence in, but not exclusive to, deep-sea settings is the genus *Lucinoma* (Salas and Woodside 2002, Oliver and Holmes 2006a, Cosel 2006, Cosel and Bouchet 2008). Oliver and Holmes (2006a) and Cosel (2006) both comment on the considerable variation in shell form observed in some species, *Lucinoma gagei* Oliver and Holmes, 2006a and *Lucinoma myriamae* Cosel, 2006 respectively. This contrasts with the relatively small morphological differences cited for the discrimination of other species, e.g. *Lucinoma kazani* (Salas and Woodside 2002) and *Lucinoma vestita* (Dautzenberg and Fischer 1906 in Cosel 2006). In the latter instances geographic isolation and habitat preferences have played a major role in the in the interpretation of the significance of morphological variation.

#### Genus Lucinoma

Dall, 1901

##### Type species.

*Lucina filosa* Stimpson, 1851

##### Definition.

As given by Oliver and Holmes (2006a)

#### Lucinoma asapheus sp. n.

urn:lsid:zoobank.org:act:E684B2EE-7C97-4FE9-9445-9B00CFE0259B

http://species-id.net/wiki/Lucinoma_asapheus

Fig. 7

##### Type material.

*Holotype*; one complete specimen, live collected, TTR 15, stn AT569GR, El Arraiche field, Mercator MV. 35°17.917'N, 06°38.717'W, 358m, 25 July 2005, NMWZ.2010.4.5.

*Paratypes*; five specimens, as holotype, NMWZ.2010.4.6.

##### Measurements (in mm)

**Table:** 

	Length	Height	Width	Anterior scar length	Anterior scar angle	Lunule width
Holotype	33.3	30.1	15.9	15.0	15°	2.2
Paratype	25.0	23.0	10.8	11.3	17°	1.6
Paratype	30.7	27.6	18.0	14.6	16°	2.6
Paratype	28.8	27.1	13.5	13.0	15°	1.7
Paratype	32.1	29.2	16.4	16.4	18°	2.6
Paratype	31.7	29.4	15.4	16.4	15°	2.2

##### Description.

**Figure 7.**
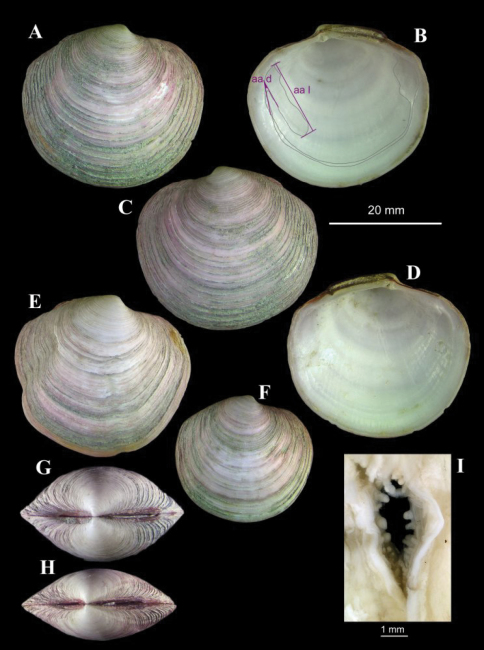
*Lucinoma asapheus* sp. n. stn. AT569GR, Mercator MV. **A–D** Holotype, aa d, angle of divergence of anterior adductor scar. aa l, length of anterior adductor scar **E** an aberrant specimen **F** a small specimen **G–H** two specimens showing variation in tumidity **I** the inhalant siphon.

(Fig. 7). Shell to 34 mm in length. Solid. Equivalve. Equilateral. Tumidity variable (Fig. 7G, H) but mostly rather compressed. Umbos low, beaks pointing forward. Outline lenticular; posterior dorsal margin almost straight, sloping gently; posterior margin curved but less so than anterior; anterior dorsal margin short, a little concave. Escutcheon narrow, edges slightly raised, extending the length of the posterior dorsal margin; three-quarters filled by ligament, remainder smooth. Lunule distinct, width dependant on tumidity of shell; edges raised, sharp. Sculpture of numerous, low but erect, thin, concentric lamellae; between lamellae are weak concentric lines. Ligament external as a prominent, raised, arched band. Set on a narrow nymph. Hinge weak; two small cardinal teeth in each valve, RV anterior and LV posterior weakly bifid; anterior lateral protuberance distinct to obscure. Pallial line entire. Anterior adductor scar greatly elongate, approximately 3/4 free from pallial line. Shell white, periostracum thin but persistent, straw coloured (all material collected has been stained in Rose Bengal, thus the pink tinge).

The anatomy is essentially that described for *Lucinoma borealis* by Allen (1958) and for *Lucinoma kazani* by Salas and Woodside (2002). The inhalant siphon is surrounded by numerous short tentacles and papillae (Fig. 7I)

##### Variation.

The shell can be rather compressed (Fig. 7H) or tumid (Fig. 7G) and this may be related to age rather than size as suggested by Oliver and Holmes (2006a) for *Lucinoma gagei* from the Arabian Sea. Some shells also show distortion with radial depressions developing abruptly (Fig. 7E).

##### Molecular data.

Tissues were sent to Dr. John Taylor (NHM, London) for inclusion in his survey of Lucinidae and the 16S and CO1 genes were compared with those of *Lucinoma borealis*. The results although not entirely conclusive indicate that the two populations are not conspecific. More recently, John Taylor’s group has demonstrated that *Lucinoma kazani* and *Lucinoma borealis* are distinct (J. Taylor pers. comm).

##### Distribution.

Only found live at Mercator MV in the Gulf of Cadiz (358m).

##### Etymology.

*asapheus* from *asaphes* Greek: meaning “indistinct” and “baffling”, referring to the lack of distinctive morphological characters and the consequent unsettling taxonomic issues.

##### Remarks.

A morphometric analysis was done comparing the Gulf of Cadiz shells with those of *Lucinoma borealis* from numerous localities from around the British Isles. This analysis could not demonstrate any statistically valid differences in the outline, the relative size of the anterior adductor scar (aa l on Fig. 7B) or the angle of divergence of this scar from the pallial line (aa d on Fig. 7B). It should be noted that the Cadiz sample size was small and that conclusive probability results were unlikely. However, the variation in tumidity and irregularity of some of the Cadiz shells is not found in samples of *Lucinoma borealis*. Anatomically *Lucinoma asapheus* and *Lucinoma borealis* are alike including the papillae that surround the inhalant aperture. Further evidence for the species level distinction between *Lucinoma asapheus* and *Lucinoma borealis* comes from the molecular data but here again the few specimens available curtails the analysis. Ecologically one might expect mud volcanoes and near shore sulphide enriched sediments to support different species. This argument was used by Salas and Woodside (2002) to support the distinction between *Lucinoma kazani* and *Lucinoma borealis*, but they also listed some morphological differences and this has been supported by molecular data (J. Taylor pers comm). Some of these, namely the tumidity of the valves, the width of the lunule and the expression of the lateral teeth are found here to be variable and therefore not conclusive. Similar variability was recorded for *Lucinoma gagei* (Oliver and Holmes 2006a) and *Lucinoma myriamae* (Cosel 2006) suggesting that small morphological differences in *Lucinoma* shells, especially if observed between small samples, may not be reliable taxonomic characters. The papillation of the inhalant siphon does appear to be much less developed in *Lucinoma kazani* compared with that in *Lucinoma borealis* and *Lucinoma asapheus*. The angle of divergence of the anterior adductor scar also shows a difference with that in *Lucinoma kazani* having a mean value of ca. 25° and both *Lucinoma borealis* and *Lucinoma asapheus* a mean value of ca. 15°. Given that *Lucinoma kazani* and *Lucinoma asapheus* both inhabit mud volcano settings one might expect them to be conspecific. However, accepting the morphological differences given by Salas & Woodside between *Lucinoma kazani* and *Lucinoma borealis* and that these also hold true for *Lucinoma asapheus* then the two must be considered distinct. This may be supported by the wide difference in depth range with *Lucinoma asapheus* coming from 358m in contrast to *Lucinoma kazani* from 1700–2030m.

Other Eastern Atlantic species are *Lucinoma vestita* (Dautzenberg and Fischer 1906) from Cape Verde at 600m, *Lucinoma atalantae* Cosel, 2006 from Mauritania at c.2000m and *Lucinoma myriamae* Cosel, 2006 from the Angola margin at c.360m. Comparisons with *Lucinoma asapheus* are as follows. *Lucinoma vestita* is a smaller species not exceeding 16mm in length, more rounded in outline and with a weak sculpture of poorly developed (often absent) comarginal lamellae. *Lucinoma atalantae* has a distinctly longer and more steeply sloping anterior dorsal margin, a more angular posterior profile and irregular sculpture. *Lucinoma myriamae* is much larger reaching over 50mm in length and has a distinct angular anterior profile; in this respect, it resembles *Lucinoma saldanhae* Barnard, 1964 a species not considered by Cosel (2006).

### Family Thyasiridae Dall, 1901

The Thyasiridae of the Gulf of Cadiz were reported on by Rodrigues et al. (2008) and of the seven species recognized only one was closely associated with active mud volcanoes, namely *Thyasira vulcolutre* Rodrigues & Oliver, 2008). Here we report upon an additional species to the Gulf of Cadiz from the Captain Arutyunov MV. Since the publication of Rodrigues et al. (2008) thyasirids from the Eastern Mediterranean mud volcanoes and the REGAB site off West Africa have become available for study. These species will not be described here but are compared with *Thyasira vulcolutre*.

#### Genus Spinaxinus

Oliver & Holmes, 2006b

##### Type species.

*Spinaxinus sentosus* Oliver & Holmes, 2006b

##### Definition.

As given by Oliver and Holmes (2006b)

#### Spinaxinus cf. sentosus

Oliver & Holmes, 2006b

http://species-id.net/wiki/Spinaxinus_sentosus

Fig. 8A–D

##### Material examined.

One live collected specimen, MSM01.03, Stn 190, deep-water field, Captain Arutyunov MV, 35°39.665'N, 07°19.970'W, 1322m, 28 April 2006, NMWZ.2010.4.7

##### Description.

**Figure 8.**
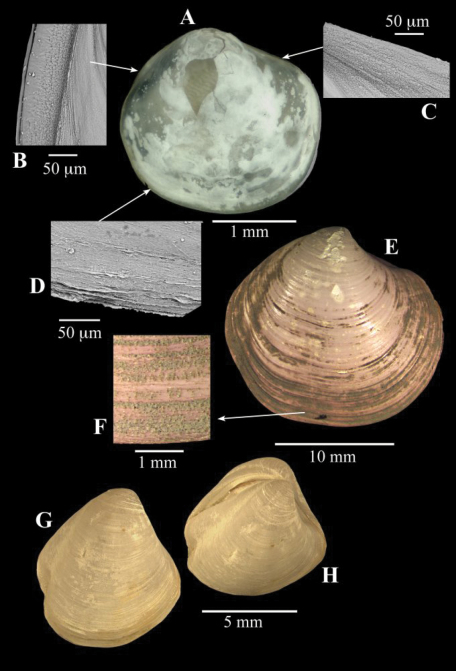
**A–D** *Spinaxinus cf. sentosus* Oliver & Holmes, 2006b. stn. 190, Captain Arutyunov MV. **A** digital image of right valve **B** SEM, periostracum on posterior margin **C** SEM, periostracum on anterior dorsal margin **D** SEM, periostracum on ventral margin. **E–F** *Thyasira* sp., Regab pock mark **E** external of right valve **F** periostracum. **G–H** *Thyasira striata*, Sturany, MEDINAUT, Eastern Mediterranean **G** external of right valve **H** oblique view showing posterior sulci.

(Fig. 8A–D). This specimen measures only 2.3 mm in length and is damaged. The outline agrees with that of *Spinaxinus sentosus* in being extended anteriorly with a long lunule depression and in the presence of a long but shallow posterior sulcus. These features are in contrast to the juveniles of *Thyasira vulcolutre* (Rodrigues and Oliver 2008), which also occurs at similar depths and settings.

The periostracal spines are typical of *Spinaxinus* but are not seen in this specimen. The periostracum is coarse and the vestiges of lamellae and projections can be seen on the extreme edges of the shell especially on the ventral margin.

##### Remarks.

Although the identification is not conclusive the likelihood that this shell is a juvenile *Spinaxinus* is high and as such represents the first finding of this species in a non-anthropogenic setting. The proximity of the type locality (off northern Portugal) to the Cadiz mud volcanoes makes this supposition more reasonable.

#### Thyasira vulcolutre

Rodrigues & Oliver, 2008

http://species-id.net/wiki/Thyasira_vulcolutre

##### Comments.

*Thyasira vulcolutre* belongs to a group of thyasirids with relatively large shells with weakly defined posterior sulci. It was concluded that it was most similar to *Thyasira southwardae* (Oliver and Holmes 2006b) from the Anyas Garden site. At that time no thyasirid material had been found at cold seep sites along the West African margin. Consequently, unlike the situation for *Lucinoma* (Cosel 2006) and *Isorropodon* (Cosel and Salas 2001, Cosel and Olu 2008) no further biogeographic comparisons could be made. Recently we were able to examine a thyasirid (Fig. 8E–F) from the REGAB site (courtesy of Karine Olu) and although it superficially resembles *Thyasira vulcolutre* it significantly differs in having a minutely spicate periostracum (Oliver in prep).

*Thyasira striata* Sturany, 1896 has long been known from deep water in the eastern Mediterranean but was recently re-discovered at cold seep sites (Olu-Le Roy et al. 2004). Small specimens resemble *Thyasira flexuosa* but larger examples (Fig. 8G–H) are very tumid with prominent lunule and deep posterior sulci quite unlike *Thyasira vulcolutre*.

These new data suggest that those thyasirids closely associated with active cold seeps have restricted ranges within the eastern Atlantic/Mediterranean region.

### Superfamily Glossoidea Gray, 1847 Family Vesicomyidae Dall & Simpson, 1901

The family Vesicomyidae has become familiar as a group of large chemosymbiotic clams associated with hot vents (Boss and Turner 1980, Tunnicliffe 1991) and cold seeps (Turner 1985, Okutani and Métivier 1986) and exemplified by *Calyptogena magnifica* Boss and Turner 1980. Not all taxa are large and the smallest, such as *Vesicomya atlantica*, are probably not chemosymbiotic (Allen 2001). Despite their conspicuous presence in many reducing environments, the taxonomy of vesicomyids is far from being settled, at both the species and supraspecific levels (Krylova and Sahling 2006). Different authors estimate that the family includes from 50 to more than 70 recent and fossil species and new species are constantly erected (Cosel and Salas 2001, Krylova and Sahling 2006, Krylova and Janssen 2006, Cosel and Olu 2009, Krylova et al. 2010). To date, fifteen chemosymbiotic species in the genera *Waisiuconcha*, *Isorropodon*, *Callogonia*, *Wareniconcha*, *Elenaconcha*, *Calyptogena*, *Christineconcha*, *Laubiericoncha* and *Abyssogena* have been reported from the eastern Atlantic (Krylova and Sahling 2010).

#### Genus Isorropodon

Sturany, 1896

##### Type species.

*Isorropodon perplexum* Sturany, 1896

##### Definition.

As given by Cosel and Salas (2001)

#### Isorropodon megadesmus sp. n.

urn:lsid:zoobank.org:act:317BA11E-0B29-4396-8696-DEDCAF00B29F

http://species-id.net/wiki/Isorropodon_megadesmus

Figs 9 10C–D

##### Material examined.

*Holotype*: one complete specimen, live collected, MSM01.03, stn 218, deep-water field, Captain Arutyunov MV. 35°39.642'N, 07°20.049'W, 1321m, 30 April 2006, NMWZ.2010.4.8.

*Paratypes*: ten specimens, four shells and one valve, same data as holotype, NMWZ.2010.4.9.

*Other material examined:* over thirty decalcified juvenile specimens, MSM01.03, stn 218, deep-water field, Captain Arutyunov MV. 35°39.642'N, 07°20.049'W, 1321m, 30 April 2006; one specimen, MSM01.03, stn 225, same locality, 35°39.707'N, 07°20.020'W, 1322m, 4 May 2006.

##### Measurements (in mm)

**Table:** 

	Length	Height	TumidityOne valve (paired)	Ratio (L/T)
Holotype	11.2	8.2	2.3 (4.6)	2.4
Paratype	14.8	11.5	3.25 (6.5)	2.3
Paratype	10.6	7.7	2.3 (4.6)	2.3
Paratype	6.2	4.4	1.3 (2.6)	2.4
Paratype	6.6	4.5	1.3 (2.6)	2.5
Paratype	9.1	6.4	1.85 (3.7)	2.5
Paratype	9.6	7.2	incomplete	

##### Description.

**Figure 9.**
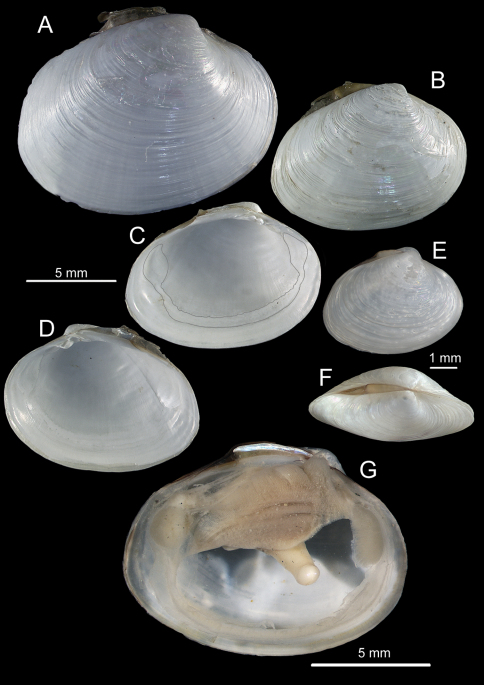
*Isorropodon megadesmus* sp. n. stn. 218, Captain Arutyunov MV. **A** large right valve, paratype **B–D** holotype, right valve external, left valve internal, right valve internal **E** small right valve, paratype **F** dorsal view, paratype **G** gross anatomy viewed after removal of right valve and mantle.

**Figure 10.**
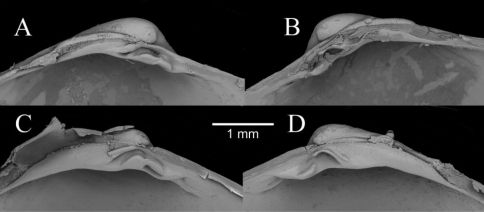
Comparison between the hinge teeth of *Isorropodon* sp. indet. (**A, B**) and *Isorropodon megadesmus* sp. n. (**C, D**).

(Figs 9, 10C–D). To 15mm in length. Thin. Equivalve. Inequilateral, beaks in front of the midline. Compressed, length to tumidity ratio 2.3 to 2.5. Outline subovate, anterior rounded, posterior a little obliquely truncated; ventral curvature at its maximum well to the posterior of the mid line. Lunule indistinct, not depressed. Escutcheon narrow, deeply excavated but entirely occupied by ligament. Sculpture of dense concentric lines and irregular growth stops or wrinkles. Hinge plate prominent dominated by a long nymph supporting a very large external ligament; ligament rises well above the dorsal margin of the shell and extends posteriorly beyond the nymph to fill the escutcheon. Hinge teeth complex; RV with a single prominent anterior lateral tooth situated in front of the beak in the form of a narrow projecting peg with a flat or slightly excavated dorsal surface; below the beak is an arched laminar tooth its anterior end overlapping the lateral tooth, its posterior slopes steeply and ventrally and merges with a second ridge only noticeable by a weak notch mid way on this combined ridge. LV with a thin laminar posterior cardinal angled obliquely plus two combined cardinals in a horizontal orientation the posterior part larger than the anterior with a distinct notch between the two parts. Pallial line entire with a very small straightened section below the posterior adductor scar; adductor scars of about equal size; anterior pedal retractor scar deeply impressed, situated immediately in front of the hinge plate. Periostracum thin, persistent, glossy. Shell white.

Mantle thin, mantle edge unfused except for short inhalant and exhalant siphonal apertures; inhalant aperture with many papillae increasing in size dorsally, exhalant with papillae of equal size. Foot with a distinct finger-like toe and poorly developed heel, pedal retractors prominent, the anterior attached in a deep impression close to the hinge. Anterior adductor muscle oval in cross-section, posterior adductor muscle subcircular, smaller than the anterior one. Ctenidia of a large, single (inner) demibranch, ascending part approximately one half the height of the outer, filaments fine tightly connected.

##### Distribution.

*Isorropodon megadesmus* is restricted to Captain Arutyunov MV (1321–1322m).

##### Etymology.

*megadesmus* from the Greek *mega* meaning large and *desma* meaning bond; referring to the external ligament.

##### Remarks.

The taxonomy of *Isorropodon* in the Atlantic and Mediterranean is complex and potentially confused (Cosel and Salas 2001, Cosel and Olu 2009). Cosel and Salas (2001) described two new species from the Eastern Atlantic, namely *Isorropodon bigoti* and *Isorropodon curtum*. They transferred a third from *Kelliella*, namely *Isorropodon elongatum* (Allen 2001). In discussing, the Mediterranean, *Isorropodon perplexum* Cosel and Salas (2001) stated that *Isorropodon* species are variable with regard to outline, tumidity and development of hinge teeth and this is illustrated in their figures 36–47 for *Isorropodon perplexum*. They noted similarities in shell morphology between the Eastern Mediterranean species *Isorropodon perplexum* and the West African *Isorropodon bigoti* but suggested that these taxa were isolated geographically and doubtfully could have gene flow between them. They further supported this argument by stating that *Isorropodon perplexum* had not been found in the Western Mediterranean or Ibero-Moroccan Gulf. In 2009, Cosel and Olu described another *Isorropodon* from West Africa (*Isorropodon atalantae*) and placed another vesicomyid in this genus (*Isorropodon striatum* Thiele and Jaeckel 1931). Therefore, before the discovery of *Isorropodon* in the Gulf of Cadiz there were already five west African species and one from the eastern Mediterranean. With the discovery of *Isorropodon* at the Capt. Arutyunov MV the assertion made by Cosel and Salas (2001) on genetic isolation can be questioned, as there is the possibility of gene flow between the seeps in the Mediterranean and around the east African coast.

In contrast to the variability given by Cosel and Salas (2001) for *Isorropodon perplexum*, all of the shells from station 218 examined here are constant with regard to features of outline, tumidity and hinge teeth. However, a single shell from station 180 is distinct, being inflated, having a distinct lunule, having a much smaller ligament and in the ventral margin being more convex. The shells from station 218 are distinct from all the shells of *Isorropodon perplexum* figured by Cosel and Salas (2001) in having a much longer nymph with the posterior teeth reaching only about one third of the ligament as opposed to the half distance given for *Isorropodon perplexum* by Cosel and Salas (2001). Furthermore, most of the shells illustrated by them have a more convex ventral margin than the shells from station 218. The single shell from station 180 shares more features with the Mediterranean shells in having the small ligament, convex ventral margin and being more inflated.

The outline of *Isorropodon bigoti* differs from all of the above in the narrower anterior and distinct angulation of the ventral curve, but is has a short nymph similar to *Isorropodon perplexum* and the shell from station 180.

*Isorropodon atalantae* has a more sunken lunule and more angular posterior profile than either of the Gulf of Cadiz taxa. *Isorropodon curtum* Cosel and Salas, 2001, from off Mauritania, is more circular in outline and *I striatum* Thiele and Jaeckel, 1931 from off Angola, is a much larger and more elongate form.

Cosel and Salas (2001) reassigned *Kelliella elongata* Allen, 2001 to the genus *Isorropodon*. Following examination of the type material in the Natural History Museum, London (BMNH 1998180) we conclude that it is not conspecific with any of the taxa discussed here. It is a small species not exceeding 2mm in any of the over 300 specimens listed by Allen (2001). It is inflated with a distinct lunule but the demarcating line illustrated by Allen (2001) is not so apparent. The hinge of the right valve has three distinct teeth including a small posterior tooth (4b in Allen 2001), which is not present in either of the species from the Cadiz mud volcanoes. Furthermore, the ligament is small and does not project as in *Isorropodon megadesmus*. From the ctenidial anatomy there is no indication that this species is chemosymbiotic. In addition to the morphological differences, *Isorropodon elongatum* has been collected from a wide geographical range, wide bathymetric range and associated with the typical oligotrophic deep-sea bivalve assemblage (derived from Allen 2008). It would appear that *Isorropodon elongatum*, if a chemosymbiotic species is not confined to seep/vent settings but as stated by Allen (2001) it is absent from the European basin and it was not present in the samples taken in the Gulf of Cadiz away from the vicinity of the mud volcanoes (Rodrigues 2009).

#### Isorropodon sp indet

Figs 10A–B 11

##### Material examined.

One complete specimen, live collected, MSM01.03, stn 180, deep-water field, Captain Arutyunov MV. 35°39.740'N, 07°19.960'W, 1323m, 27 April 2006, NMWZ.2010.4.10.

##### Measurements.

6.4mm (L) × 5.3mm (H) × 3.6mm (T)

##### Description.

**Figure 11.**
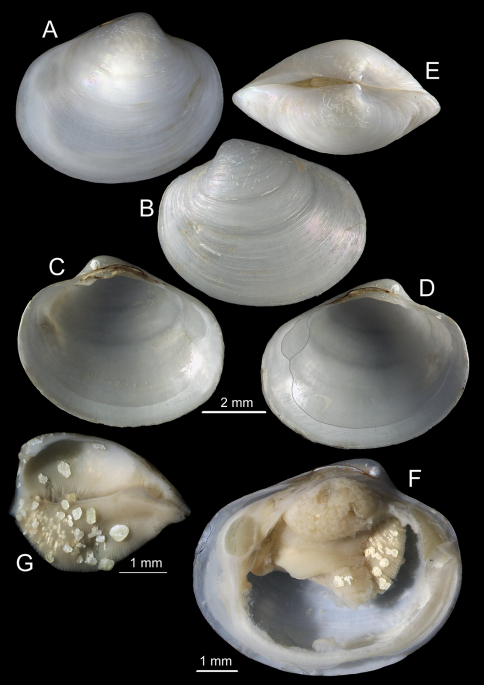
*Isorropodon* sp. indet., stn. 180 Captain Arutyunov MV. **A–D** External and internal views of right and left valves **E** dorsal view **F** gross anatomy viewed after removal of right valve and mantle **G** Excised ctenidium with crystalline artifacts.

(Figs 10A–B, 11). 6.4 mm in length. Thin. Equivalve. Inequilateral, beaks in front of the midline. Inflated, length to tumidity ratio = 1.8. Outline subovate, anterior bluntly rounded, posterior a little obliquely truncated; ventral curvature at its maximum more or less at the midline. Lunule indistinct, slightly depressed. Escutcheon narrow, deeply excavated. Sculpture of dense, concentric, fine lines and few irregular growth stops. Hinge plate narrow, nymph supporting an external ligament; ligament scarcely rises above the dorsal margin of the shell and extends posteriorly to half the length of the escutcheon. Hinge teeth complex; RV with a single prominent anterior lateral tooth situated in front of the beak in the form of a narrow projecting peg with a flat or slightly excavated dorsal surface; below the beak is weakly arched laminar tooth its anterior end overlapping the lateral tooth, its posterior slopes steeply and ventrally and merges with a second ridge only noticeable by a weak notch mid way on this combined ridge. LV with a thin laminar posterior cardinal angled obliquely plus two combined cardinals in a horizontal orientation the posterior part only slightly larger than the anterior with a distinct notch between the two parts. Pallial line entire with a very small straightened section below the posterior adductor scar; adductor scars of about equal size; anterior pedal retractor scar deeply impressed, situated immediately in front of the hinge plate. Periostracum thin, persistent, glossy. Shell white

Mantle thin, mantle edge unfused except for short inhalant and exhalant siphonal apertures; inhalant aperture with few papillae increasing in size dorsally, the latter as short tentacles, exhalant with papillae of equal size. Foot with a blunt finger-like toe and poorly developed heel, pedal retractors prominent, the anterior attached in a deep impression close to the hinge. Anterior adductor muscle pyriform in cross-section, posterior adductor muscle subcircular, smaller than the anterior one. Ctenidia of a large, single (inner) demibranch, filling the majority of the mantle cavity, ascending part approximately one half the height of the outer, filaments fine tightly connected.

The numerous crystalline growths seen on and between the filaments are believed to be natural and not an artifact of preservation.

##### Distribution.

*Isorropodon* sp indetis restricted to Captain Arutyunov MV (1323m).

##### Remarks.

As discussed above for *Isorropodon megadesmus*.

## Ecological Discussion

### Symbiosis.

Twelve bivalve species have been found in close association with chemosynthetic settings in the Gulf of Cadiz (this paper; Génio et al. 2008, Rodrigues et al. 2008, Rodrigues et al. 2010) with a thirteenth, *Callogonia cyrili* Cosel and Salas probable but not proven (Cosel and Salas 2001). The trophic status of these Solemyidae, Lucinidae, Thyasiridae and Vesicomyidae species has been confirmed by their gross anatomical features (e.g. gills for thyasirids, reduced gut for solemyids) and, in some cases, also by stable isotope analysis and/or molecular analysis (Rodrigues et al. 2010, Rodrigues and Duperron 2011). The δ13C values for solemyids (*Acharax gadirae*, *Solemya* (*Petrasma*) *elarraichensis*), lucinid (*Lucinoma asapheus*) and thyasirid (*Thyasira vulcolutre*) bivalves were found to be in line with data for other bivalves known to host thiotrophic symbionts (Fisher 1990, Carlier et al. 2010). On the other hand, δ13C and δ34S values for the bathymodiolid species (*Bathymodiolus mauritanicus*) were compatible with the predominance of methanotrophy. Phylogenetic analysis of bacterial 16S rRNA gene sequences demonstrated that most bacteria were related to known sulfide-oxidizing endosymbionts found in other deep-sea chemosynthetic environments, with the co-occurrence of methane-oxidizing symbionts in *Bathymodiolus* specimens. The molecular results confirmed the thiotrophic nutrition for *Solemya (Petrasma) elarraichensis*, *Acharax gadirae*, *Lucinoma asapheus* and *Thyasira vulcolutre* and a dual symbiosis for *Bathymodiolus mauritanicus* (Rodrigues et al. 2010). Nutrition of *Isorropodon megadesmus* was not yet confirmed by either isotopic or molecular analyses but other studies suggest that *Isorropodon perplexum* contain sulphur-oxidizing bacteria (Olu-Le Roy et al. 2004), a phylotype also present in *Spinaxinus sentosus* (Oliver and Holmes 2006b).

### Distribution patterns.

The Gulf of Cadiz mud volcano field is comprised of over thirty seeps of various activity and spread over a bathymetric range of 200–4000m. Of the 25 mud volcanoes sampled, 13 have chemosymbiotic species, which indicates their importance in the structure of the seep assemblages (Fig. 1). Most of the thirteen, chemosymbiotic, species found in the Gulf of Cadiz are restricted to one or two mud volcanoes. This patchy distribution can result from physical or physiological constraints such as depth, distance and fluid flow rates. They were more frequent in the shallower mud volcanoes (200–1500m) but were especially diverse in Captain Arutyunov MV where five different species co-occur (Fig. 1). Some taxa are confined to single mud volcanoes whereas others are more widespread.

The family most frequently encountered in the chemosynthesis-based assemblages of the mud volcanoes from the Gulf of Cadiz, is the Solemyidae. The family is represented by two genera, *Solemya* (*Petrasma*) with a shallower distribution (358–1030m) and *Acharax* with a deeper distribution (556–3902m) but co-occurring at intermediate depths in the Western Moroccan field. Co-occurrence of these genera has not been reported elsewhere and may be explained by the apparent absence of the subgenus *Petrasma* from seep settings preferring reducing sediments and low oxygen conditions (Kamenev 2009).

This is the first record of this family in cold seeps from the North-east Atlantic, although *Solemya* (*Solemya*) *togata* is well known from shallow settings such as sea-grass beds in the Mediterranean. Why this species has not or been unable to colonize the shallow mud volcanoes is unknown. In contrast, the Pacific *Solemya (S.) tagiri* Okutani et al. (2003) is thought to be associated with seep settings (Kamenev 2009) suggesting that habitat is not linked to phylogeny.

Unlike *Solemya* (*Petrasma*), *Acharax* species are consistently associated with seep or vent settings and some species such as the Pacific *Acharax johnsoni* have extensive bathymetric ranges from 100 to over 5000m (Kamenev 2009). *Acharax* species are all rather morphologically similar and given the molecular data given by Neulinger et al. (2006) it may be found that there are more species than currently recognized. This is suggested here by the morphological differences in the posterior aperture between the shallow and deeper specimens. Some *Acharax* specimens (from Yuma, Ginsburg and Jesus Baraza MVs) host commensal polychaetes (Ravara et al. 2007).

In contrast to the solemyids, the lucinid *Lucinoma asapheus* has only been collected at Mercator MV, although video observations revealed presence of lucinids in other mud volcanoes from the Spanish field (MR Cunha, pers. comm.). *Lucinoma asapheus* is very similar morphologically to *Lucinoma kazani* from the Eastern Mediterranean (Salas and Woodside 2002) and both are almost identical with the widespread shelf species *Lucinoma borealis* that is abundant in the North-east Atlantic. Our preliminary molecular data separated *Lucinoma borealis* from *Lucinoma asapheus*, and further work on *Lucinoma kazani* confirms that it is also distinct from *Lucinoma borealis* (J Taylor, pers comm.). A more detailed study including a wider data set from *Lucinoma asapheus* should elucidate the relationship between it and *Lucinoma kazani*. The three species so far mentioned are similar but quite distinct from the much larger, deeper water SE Atlantic species such as *Lucinoma myriamae* and *Lucinoma saldanhae* and the Indo-Pacific species, *Lucinoma gagei* and *Lucinoma yoshidai* (Oliver and Holmes 2006a). It is therefore plausible that *Lucinoma asapheus, L. kazani* and *Lucinoma borealis* are a clade, but we cannot ascertain their sequence of appearance. Are the mud volcano species independently evolved from shallow water ancestors or are they descended from a common seep dwelling stock?

The thyasirid *Thyasira vulcolutre* was only found in the deep-water mud volcano field (Rodrigues et al. 2008). Despite the high number of thyasirid species present in the Gulf of Cadiz (eight) only one *Thyasira vulcolutre* is strictly associated with active mud volcanoes (Rodrigues et al. 2008). *Spinaxinus sentosus* has been discovered in the organic cargo of a sunken ship in the Atlantic Ocean (Oliver and Holmes 2006b) and is reported here for the first time associated to a cold seep site. Other chemosymbiotic thyasirids (Table 1) were found associated with Siboglinidae fields from the Hakon-Moseby MV (Gebruk et al. 2003) and from the eastern Mediterranean mud volcanoes (Olu-Le Roy et al. 2004).

Small vesicomyids including *Isorropodon megadesmus* and *Isorropodon sp.* were very abundant in Captain Arutyunov MV.The species *Isorropodon perplexum* is known only from the Eastern Mediterranean (Table 1), and was shown to harbour sulphur-oxidizing bacteria (Salas and Woodside 2002). During TTR17 (2008) a living specimen of *Calyptogena* was found for the first time in the Gulf of Cadiz. The specimen collected from Bonjardim has been identified as belonging to the species *Christineconcha regab* Cosel and Olu, 2009, recently found in the Regab region (E Krylova, pers. comm.). Other large vesicomyid shells but no living specimens of *Laubiericoncha chuni* (Thiele and Jaeckel 1931) and *Pliocardia* sp. were also collected at the same mud volcano. The vesicomyids *Calyptogena regab* and *Lucinoma chuni* dominate the faunal assemblages of Congo and Angola Basin cold seeps (Olu-Le Roy et al. 2007, Cosel and Olu 2008).

Bathymodioline mussels of the amphi-Atlantic species *Bathymodiolus mauritanicus* were only found living in Darwin MV although extensive graveyards of mussel shell ash are also found in other mud volcanoes of the western Moroccan field (Génio et al. 2008). Although confirmed as chemosymbiotic, *Bathymodiolus mauritanicus* was first collected during a commercial trawl in the Mauritanian margin where no hydrocarbon seeps have yet been discovered. Nevertheless owing to the repeated appearance of other typical seep molluscs it is likely the existence of seepage in this region might be confirmed in the future (Cosel 2002). Another small mytilid *Idas* sp. was collected living in a small wood fall retrieved by the ROV Isis at Carlos Ribeiro MV during JC10. *Idas* specimens are present in the mud volcanoes from the Eastern Mediterranean (Table 1) and their symbioses have been studied in detail by Duperron et al.(2008).

When discussing *Isorropodon*, Cosel and Salas (2001) suggested that Mediterranean and SE Atlantic forms were unlikely to be the same species due to their geographic isolation. With the discovery of *Isorropodon* in the Gulf of Cadiz, this argument can be challenged. Here we review the distributions of all chemosymbiotic taxa found at seeps in the Eastern Atlantic/Eastern Mediterranean and consider the apparent levels of endemism. Table 1 presents the known taxa and their occurrence in the four major seep fields, namely Eastern Mediterranean, Gulf of Cadiz, off Mauritania and the Gulf of Guinea.

The Gulfs of Cadiz and Guinea are most diverse and almost equally so with 13 and 14 species respectively. The less sampled Mauritania basin has only 7 recorded species while the well studied Eastern Mediterranean has only 6 species. The number of species common to more than two fields is zero and the maximum number of shared species is two, that for the Gulfs of Cadiz and Guinea. These data suggest high levels of endemism within fields but where there is overlap, especially with the larger vesicomyids, that this occurs at deeper sites. The vesicomyids are the most diverse family but only one species has colonized the Eastern Mediterranean and they appear to be rare in the Gulf of Cadiz compared with the Gulf of Guinea. From a geological history perspective one can explain the poor diversity in the Eastern Mediterranean from the shorter period of time for colonization since the re-invasion of Atlantic waters post the hyper-saline event. At this time there is no evidence to indicate the origins of the Eastern Atlantic faunas, either by dispersal or local speciation. Molecular data from the species rich genus *Isorropodon* may illuminate the relationships and sequence of speciation and we await the study in progress on the Vesicomyidae mentioned by Cosel and Olu (2008). Warén and Bouchet (2009) in discussing seep gastropods stated that the West African fauna was quite distinct from that of the Gulf of Cadiz at both species and generic levels and suggested that the latter was of more recent origin via local radiation. At the generic level, these findings appear at odds with the bivalve data where there are strong similarities between the Gulfs of Guinea and Cadiz. The exceptions are *Solemya (Petrasma)*, which appears to have a NW Atlantic origin and *Lucinoma* species, where their origin may be local.

Warén and Bouchet (2009) further indicated strong affinities of the West African fauna with the Western Atlantic and Gulf of Mexico faunas. Relationships among the bivalves with the western Atlantic at the species level are few, if any; only with *Bathymodiolus mauritanicus* is there a suggestion of an amphi-Atlantic distribution (Génio et al. 2008). At the generic level there is considerable over-lap within the solemyids, lucinids and thyasirids but less so with the vesicomyids. This may be in part to the considerable number of new genera erected for eastern Atlantic taxa by (Cosel and Olu (2008, 2009).

**Table 1.** Distribution of chemosymbiotic taxa known from seep/mud volcano fields in the Eastern Atlantic and Mediterranean.

Taxon	East.Mediterranean	Gulfof Cadiz	MauritaniaBasin	Gulf ofGuinea
Solemyidae				
*Acharax* indet.				X
*Acharax gadirae*		X		
*Solemya (Petrasma) elarraichensis*		X		
Solemyidae Eastern Med	X			
Mytilidae				
*Bathymodiolus mauritanicus*		X	X	
*Bathymodiolus aff. boomerang*				X
*Idas modiolaeformis*	X			
*Idas* sp.	X	X		
Lucinidae				
*Lucinoma asapheus*		X		
*Lucinoma kazani*	X			
*Lucinoma atalantae*			X	
*Lucinoma myriamae*				X
*Myrtea amorpha*	X			
*Graecina karinae*				X
*Joellina dosiniformis*				X
Thyasiridae				
*Thyasira vulcolutre*		X		
*Thyasira striata*	X			
*Thyasira* sp. n.				X
*Spinaxinus sentosus*		X		
Vesicomyidae				
*Isorropodon perplexum*	X	X?		
*Isorropodon megadesmus*		X		
*Isorropodon bigoti*			X	X
*Isorropodon curtum*			X	
*Isorropodon striatum*				X
*Isorropodon atalantae*				X
*Callogonia cyrili*		X		
*Callogonia mauritanica*			X	
*Callogonia valdiviae*			X	X
*Christineconcha regab*		X?		X
*Wareniconcha guineensis*				X
*Elenaconcha guiness*				X
*Laubiericoncha chuni*		X?		X
*Pliocardia* sp.		X		
*Abyssogena southwardae*			X	
Total( ) shared species	6 (1?) (0) (0)	(1?) 13 (1) (2)	(0) (1) 7 (2)	(0) (1) (2) 14

## Supplementary Material

XML Treatment for Petrasma

XML Treatment for Solemya (Petrasma) elarraichensis

XML Treatment for Acharax

XML Treatment for Acharax gadirae

XML Treatment for Lucinoma

XML Treatment for Lucinoma asapheus

XML Treatment for Spinaxinus

XML Treatment for Spinaxinus cf. sentosus

XML Treatment for Thyasira vulcolutre

XML Treatment for Isorropodon

XML Treatment for Isorropodon megadesmus

XML Treatment for Isorropodon
